# Inside-Out Control of Fc-Receptors

**DOI:** 10.3389/fimmu.2019.00544

**Published:** 2019-03-21

**Authors:** Leo Koenderman

**Affiliations:** Department of Respiratory Medicine and Laboratory of Translational Immunology, University Medical Center Utrecht, Utrecht, Netherlands

**Keywords:** inside-out control, immunoglobulins, priming, activation, phagocytes, Fc-receptors

## Abstract

Receptors recognizing the Fc-part of immunoglobulins (FcR) are important in the engagement of phagocytes with opsonized micro-organisms, but they also play a major role in the pathogenesis of chronic inflammatory diseases. Different FcRs are specifically recognizing and binding the different classes of immunoglobulins, transmitting different signals into the cell. The function of IgG (FcγR's) and IgA (FcαR) recognizing receptors is controlled by cellular signals evoked by activation of heterologous receptors in a process generally referred to as inside-out control. This concept is clearly described for the regulation of integrin receptors. Inside-out control can be achieved at different levels by modulation of: (i) receptor affinity, (ii) receptor avidity/valency, (iii) interaction with signaling chains, (iv) interaction with other receptors and (v) localization in functionally different membrane domains. The inside-out control of FcRs is an interesting target for novel therapy by therapeutical antibodies as it can potentiate or decrease the functionality of the response to the antibodies depending on the mechanisms of the diseases they are applied for.

## Introduction

Immunoglobulins have evolved during evolution as a link between the antigen-specific adaptive immunity and the molecular pattern driven innate immune system. These molecules contain an antigen-specific variable region brought about by gene rearrangement in B-cells. The underlying mechanisms for this rearrangement has been reviewed elsewhere (1). For the purpose of this review it suffices to say that this variable part enables specific binding to antigens beyond the common patterns recognized by the innate immune system. After the antibody binds the antigen via the variable regions, the constant region of the immunoglobulins denoted as Fc part can be recognized by other immune cells and facilitate the immune response (2–4).

The Fc-part of immunoglobulins is relevant at three different levels. First of all the Fc-part determines the human (sub)class of the immunoglobulin: IgA (IgA1 and IgA2), IgD, IgE, IgG (IgG1, IgG2a, IgG2b, IgG3, and IgG4) and IgM. During gene rearrangement the B-cell determines, guided by cytokines in its environment, which (sub)class of immunoglobulin is later produced by the respective plasma cells. The second level is the propensity of some classes of immunoglobulins to activate and fixate complement, which greatly enhances the recognition of antigens through recognition of C3b and C3bi by complement receptors (CR1/CD35 and CR3/CD11b) on phagocytic cells. The binding of immunoglobulins and complement fragments to antigens is generally referred to as opsonization.

Finally, the third level by which the Fc-part of immunoglobulins is important, is the recognition by specific Fc-receptors. These Fc receptors are mainly expressed by effector cells of the innate immune response [for excellent reviews see (2–7)]. Every class of immunoglobulins has specific receptors that can recognize these subclass specific Fc portions. These receptors are indicated by Greek letters: FcγR for IgG, FcαR for IgA, FcεR for IgE, FcδR of IgD and FcμR for IgM. Apart from these receptors also the neonatal FcR (FcRn) is expressed by stromal cells and is involved in transfer of immunoglobulins from blood to the tissue (8). Some of the immunoglobulins have more receptors with various affinities for the different subclasses as IgG comes in 5 subclasses (IgG1, IgG2a, IgG2b, IgG3, and IgG4) and IgA in two (IgA1 and IgA2). The situation with IgA is even more complex as the molecules are found as both monomers and dimers, and on mucosal surfaces as dimers with a J-chain and secretory component. The latter form of IgA is referred to as secretory IgA, which can still be recognized by FcαR (2, 3). However, additional receptors for the secretory component can modify the binding characteristics of secretory IgA (9).

The FcRs are under tight control as the immune system should evoke a balanced response to invading micro-organisms as well as to signals that can lead to aberrant activation of the immune system such as seen in chronic inflammatory disease including autoimmune disorders (10). Too much activation leads to collateral damage to the host tissue, whereas too little activation can lead to infections. The control of the function of the FcRs is the subject of this review.

## Fc-Receptor Functioning in the Innate Immune Response

The best known function of FcRs is their role in phagocytosis and killing of opsonized targets. Phagocytosis refers to the process of specialized cells of the immune system that can engulf and take up targets into intracellular organelles called phagosomes (11). These phagosomes are closed and do not have any link with the extracellular milieu. In these organelles the cells can induce a very hostile environment by which the phagocytosed target is killed. This is mediated by multiple processes: fusion of granules filled with cytotoxic proteins, enzymes and peptides, production of toxic oxygen intermediates by a membrane bound NADPH-oxidase, and a lowering of the pH in the phagosome (12).

The fusion of the granules with the phagosome is often referred to as degranulation. This fusion of the phagosome with the granules leads to the formation of so-called phagolysosomes in which the actual killing of microbes takes place. Degranulation is not only into these phagolysomes, but occurs also by fusion of the granules with the plasma membrane. Then the cytotoxic components are liberated into the extracellular space, where they are involved in killing of the targets outside the cell. It will be clear that this extracellular process comes with a cost: damage to the healthy host tissues (13). This process of extracellular killing is also employed by eosinophils and macrophages killing large multicellular targets such as helminths; targets several times larger than the immune cells. Patnode et al. (14) describe clear swarming behavior of eosinophils interacting with helminths that leads to a “together we are strong” type of killing. There is a clear synergism in killing mediated by degranulation and the activation of the NADPH-oxidase; the other major mechanism involved in killing of micro-organisms by phagocytes (15).

It will be clear from the above that FcRs are very important in the interaction of the host with pathogens. This review will focus on two classes of FcRs as these are important in phagocytosis and killing of micro-organisms: FcγR and FcαR. Six genes encode FcγR's in humans: FcγRI (CD64), FcγRIIA (CD32A), FcγRIIB (CD32B), FcγRIIC (CD32C), FcγRIIIA (CD16A), and FcγRIIIB (CD16B) (4). These receptors are expressed by various immune cells in different combinations and have different affinities for the different IgG subclasses (4). There are several IgA receptors: FcαRI (CD89), transferrin-receptor-1 (CD71), asialoglycoprotein-receptor (ASGPR/), Fcα/μR, FcRL4, and DC-SIGN/SIGNR1 (2). However, the best studied in the context of immune function and phagocytosis is FcαRI (CD89) and, therefore, we will focus on this IgA-receptor in this review.

### Signal Transduction

Signal transduction of FcRs has been studied in detail and reviewed by Bournazos et al. (16). In short, broadly three modules of signaling are found for these receptors: (1) Direct signaling by the receptor itself (CD32s), (2) Via an accessory common FcRγ-chain (CD64 and CD16A and CD89), and (3) indeterminate signaling because of the absence of an intracellular tail [Glycosylphosphatidylinisotol (GPI) anchored CD16B].

### Direct Signaling

Direct signaling by CD32 is mediated by immunoreceptor tyrosine-based activation motif (ITAM/CD32A and CD32C) (17) and by immunoreceptor tyrosine-based inhibitory motif (ITIM/CD32B) (18). These motifs determine whether the receptors are activating or inhibitory. It is important to emphasize that signaling starts by cross-linking of the receptor leading to activation of phosphatases such as SHP and SHIP, and members of the src-family of tyrosine kinases (19–21). This leads to phosphorylation of the important tyrosine residues in the ITAM/ITIM motifs from where various signaling cascades are initiated. Phosphorylation of ITAMs lead to activation of the cells (22), whereas phosphorylation of ITIMs lead to cell inhibition (23). The mechanisms involved in the control of CD32B have been excellently reviewed by Getahun and Cambier (24).

### Signaling via an Accessory Common FcRγ-Chain

Signaling via an accessory common FcRγ-chain is also mediated by ITAM motifs present in the γ-chain. Here the main signaling is not mediated by the intracellular tail of the FcR itself, but by the FcRγ-chain that is associated with the receptor. This mode of action is found for CD16A, CD64, and CD89. Similar signals are initiated compared to direct signaling from the receptor (25–27).

### Indeterminate Signaling

Indeterminate signaling seems to be the characteristic of CD16B expressed at high levels on human neutrophils. This receptor lacks both an intra-cellular portion and a transmembrane domain as it linked with the membrane with a GPI-linkage (28). However, it is likely too simple to consider this receptor as signaling dead. Various studies indicate that cross-linking CD16B evokes signaling characterized by e.g., changes in intracellular free Ca^2+^ ([Ca^2+^]_i_) (29). The general idea is that cross-linking leads to an engagement with other receptors that in turn activate a signaling cascade. The identity of such a receptor in CD16B signaling remains to be defined, but studies indicate that integrins and integrin associated proteins might be candidates(30). Such mechanism *in trans* can also be part of signaling through the other signaling FcRs (30, 31). This paradigm will be discussed in more detail later in the review.

Most of the IgG and IgA receptors exhibit a low or intermediate affinity for their monovalent ligands with an exception for FcγRI/CD64 that has a high affinity for monomeric IgG. The low affinity receptors do not bind to monomeric ligand or this binding is so low affinity that it is difficult to determine *in vivo* (32). The consequence of this low affinity is that these receptors only bind to multivalent ligand such as found in immune complexes as well as Ig coated surfaces such as found on opsonized micro-organisms(3). This in contrast to FcγRI that is always bound to IgG, but that interestingly does not lead to appreciable signaling (33).

An additional mode of control of FcRs is the multimerisation of the receptor into clusters at the cell membrane by which their valency increases (34). Modulation of this valency is a means by which the cell can facilitate the interaction with Ig-coated surface.

## The Concept of Inside-Out Control

### The Concept of Inside-Out Control Identified in Integrin Function

The concept of inside-out control of immune receptors was first put forward for the function of integrins (35). It basically refers to an increase in receptor affinity, valency and/or function induced by intracellular signals initiated by heterologous stimuli. A very clear example is the finding that a mutation of the Kindlin-3 gene in patients with leukocyte adhesion deficiency III leads to a complete block in the functionality of β2 chain containing integrins LFA-1, Mac-1 and p150.95 (36). The genes and expression of these receptors are normal, but functionality is lacking leading to a clinical phenotype reminiscent of LAD1 where the β2-chain (CD18) gene is mutated and expression of the CD18 integrins is absent (37). A similar situation is found for the fibrinogen receptor (αIIb/β3) that is dysfunctional in these Kindlin-3 deficient patients. The molecular mechanisms underlying inside-out control of integrins is excellently reviewed by the group of Ginsberg et al. (35, 38).

### Inside-Out Control of FcR

Next to integrins various studies show that also FcγR's and FcαR are subjected to inside-out control (39–43). In contrast to integrins where a consensus is present that this mechanism is important, this concept has not yet been generally accepted for FcR function. The main problem with the latter receptors is that many immune cells express multiple FcRs for the same ligand Ig which makes the study of individual receptors difficult. The studies that have focused on inside-out control of specific FcRs have either been performed with cells endogenously expressing only a single Fc-receptor or cell models dependent on cytokines exogenously expressing single Fc-receptors (39–42, 44).

### FcγRII

An excellent cell to study the inside-out control of FcγRIIA is the human eosinophil. This cell isolated from the blood of healthy control only expresses this FcγR. Early work showed that eosinophils carefully isolated in a non-primed fashion hardly bind beads coated with human IgG while they clearly express FcγRII as visualized in FACS based assays (42). Short term pre-incubation with cytokines such as IL-5 and GM-CSF or chemotaxins such as platelet-activating factor (PAF) lead to clear binding of the cells to these Ig-coated particles, whereas the expression of the receptor on the cell surface was unaltered. This model also allowed the manipulation with different pharmacological inhibitors to find out which signaling models are important in this inside-out control (44). These experiments identified that the MEK-MAP-kinase based signaling in these cells is important as MEK inhibitors clearly block the interaction of pre-activated eosinophils with Ig-coated particles (44). These findings basically imply that different cytokines differentially engaging different signaling pathways can steer the inside-out control of FcγRII: those that engage MEK-MAPK such as IL-5 steer the function of FcγRII, whereas those that more engage PI-3K and p38 such as IL-4 more activate FcαR [see below and (44)]. Similar experiments are very difficult to perform with neutrophils because of the high co-expression of FcγRIII (CD16B). It should be emphasized that Huizinga et al. have shown that FcγRII is also the main signaling IgG-receptor in neutrophils (45) and most likely controlled by a similar signaling module as operational for FcαR (42). However, direct experimental proof is lacking. Interestingly, Aleman et al. (46) described the importance of FcγRIIIB in netosis of neutrophils supporting the concept of FcγRIIIB as a signaling receptor.

### FcαRI

This receptor is expressed by multiple immune cells including eosinophils. It is, however, important to mention that FcαRI on eosinophils is heavily glycosylated and behaves differently in SDS-PAGE gels when compared with the receptor present in e.g., neutrophils (47). Comparable with serum-IgG coated beads, only (cytokine) primed eosinophils interact with IgA-coated beads (44). However, for FcαR mediated interaction between IgA-coated targets and primed eosinophils the PI-3-kinase signaling pathway is important. This has important consequences as cytokines such as IL-4 that primarily engage this pathway without apparent activation of the MAP-kinase pathway only induce binding of eosinophils with IgA coated targets and not IgG coated targets (44). Interaction with IgG coated beads is not sensitive for (cytokine) priming, likely because FcγRIIIB that is highly expressed by neutrophils can facilitate the interaction with IgG coated beads.

These findings have consequences *in vivo* as differential priming with different mediators at different times and places will determine whether innate immune cells will engage with opsonized particles. It is important to emphasize that eosinophils isolated from patients with allergic diseases exhibit a primed phenotype with respect to binding to IgG and IgA coated beads (48). This implies that these cells have engaged with Th2 driven cytokines and other mediators leading to long term priming of the cells as the primed phenotype persisted during the whole isolation procedure *ex vivo*. Thus, the FcRs retain their primed phenotype for a long time *in vitro*. The situation *in vivo* is less clear as the group of Chilvers et al. put forward the hypothesis that part of the primed phenotype of granulocytes associated with primed FcRs deprimes in the lung *in vivo* (49, 50). This concept, however, has been tested for neutrophils but not for eosinophils. The expression of multiple FcRs on neutrophils precludes a simple testing of the hypothesis that depriming leads to deactivated FcRs on granulocytes.

The mechanisms underlying inside-out control are multiple, complex and cross-interacting. They can be at the level of the receptor itself, associated signaling partner molecules, clustering of homologous and heterologous receptors allowing activation in *trans* and last but not least changes in organization of plasma membrane specialized areas such as lipid rafts and caps.

**Figure 1:**
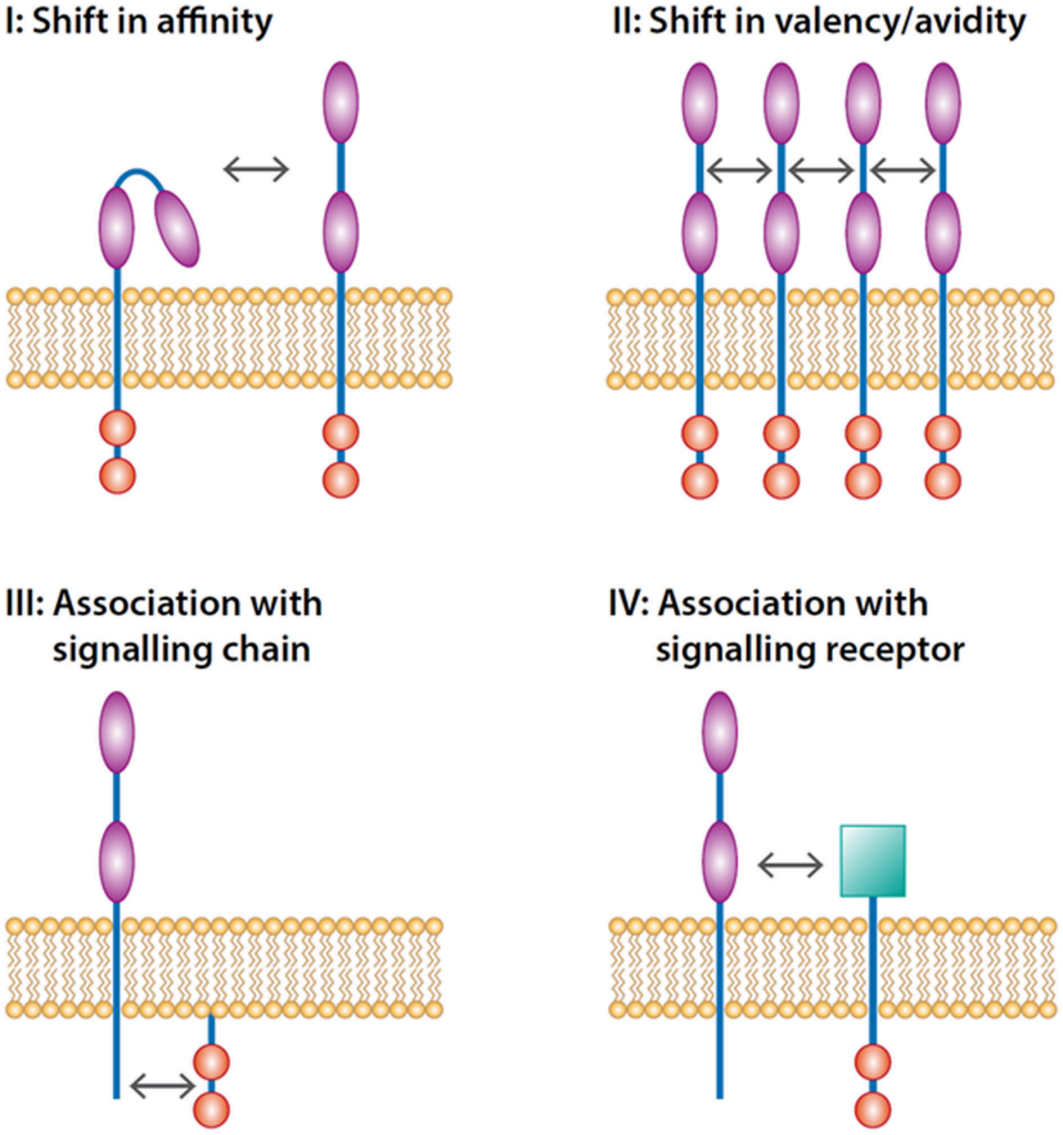
Levels of inside-out control of FcRs. Inside-out control can be accomplished at four levels. The first level is modulation of affinity of single receptors opening up the binding domain(s) of the immunoglobulin domains (illustrated by 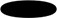) facilitating the activation of the receptor which in turn signals through signaling motifs (illustrated by 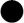) present in the receptor. The second level is induction of lateral movement of the receptor increasing the valency of the receptor. The third level is the interaction with signaling chains such as FcRγ-chain that transmit the signal-tranduction pathways. The fourth level is the trans-activation through an associated heterologous signaling receptor (illustrated by 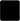).

The functionality of FcRs expressed on the plasma membrane can be accomplished at different levels: (1) changes in valency (multiple receptors are engaged by multivalent ligands on opsonized surfaces (see Figure 1), and (2) changes in affinity of single receptors for their ligands.

The valency of receptors is very important as the consensus in the field is that cross-linking of receptors by multiple ligands on the opsonized surface is the main trigger for activation through FcRs (34). It is generally believed that tyrosine kinases binding the one FcR cross-phosphorylate tyrosine residues in ITAM's/ITIM's of the adjacent FcRs. This then initiates the signaling cascades leading to the activation of the downstream functions. So these receptors have to come together in order to be able to signal. Cross-linking by itself seems to be sufficient for signaling as artificial cross-linking by receptor antibodies leads to phosphorylation of the receptors and induction of signaling (29, 51). However, artificial cross-linking does not completely recapitulate the activation induced by natural ligand. This is nicely illustrated by the finding that cross-linking of FcγRIIIB (CD16B) that does not have any intracellular tail leads to changes in intracellular free Ca^2+^ ([Ca^2+^]_i_) whereas no signaling motif is present in this receptor. Although it might be that cross-linking of FcγRIIIB engages FcγRIIA through the Fc-portion of the CD16 antibody. In addition, such changes in [Ca^2+^]_i_ are not necessarily induced by natural ligand in the form of immune complexes (45) or serum-opsonized particles (52). Thus, caution should be taken to apply artificial cross-linking of the receptor as surrogate for FcR signaling. It is also difficult to test the hypothesis that an increase in valency (receptor clustering) is sufficient for FcR signaling as it is difficult to accomplish this without additionally affecting the receptor affinity for its ligand.

The affinity of low-affinity receptors for their ligands is difficult to determine as monomeric ligand does not bind with sufficient affinity even after inside-out activation. This makes sense as the immune system ideally does not want to interact with monomeric Ig's in blood and mucosal tissues. Therefore, it is very difficult to study valency and affinity of FcRs as two independent mechanisms and, therefore, the functionality of FcR in the remainder of this review is the resultant of both increased valency and affinity.

## The Importance of the Intracellular Tail of FcRs in Inside-Out Control

In order for inside-out control to be affecting the functionality of FcRs these signals should converge at the intracellular tail of the FcRs such as also found for integrins (38). This concept is studied in more molecular detail for two FcRs: FcαRI (CD89) and FcγRI (CD64) (39–41, 53, 54).

### The Importance of Serine263 in the Intracellular Tail of FcαRI

Initial studies indicated that several kinases, PI-3-kinase, MAP kinase and p38 were critically involved in the activation of the functionality of FcαRI (40, 41, 48). This was found both in primary cells (leukocytes) as well as cell lines ectopically expressing this receptor. Inhibitors of these kinases modulated the activation FcαRI even in the absence of the accessory common Fcγ-chain. This implies that phosphorylation is involved in the direct control of the functionality of FcαRI. However, there were no clear consensus motifs present in the intracellular tail that are preferentially phosphorylated by any of these kinases. In depth analysis of the receptor led to the conclusion that FcαRI expressed in resting cells was constitutively phosphorylated and thus that kinase activity was already found in the cytosol of resting cells (41). This led to the concept that FcαRI is actively suppressed in its function by constitutive phosphorylation of the receptor. By studying FcαRI receptor mutants transfected into cytokine-dependent Ba/F3 cells it was found that a serine residue at the 263 position in the intracellular tail is essential for the functionality of FcαRI in the context of binding to IgA-coated beads. Mutation of the serine residue to alanine led to a constitutively active receptor supporting the concept that an active kinase is important in keeping the receptor in a non-functional state. This hypothesis was supported by the finding that the S>D mutation, introducing a negative charge at the 263 position, lead to a non-functional receptor as if it was constitutively phosphorylated (41).

Ten Broeke et al. recently provided evidence that the identity of this constitutively active kinase is glycogen synthase kinase-3 (GSK-3) (43), a kinase that is constitutively active in resting cells such as leukocytes and its activity is inhibited by phosphorylation (43). Interestingly, such phosphorylation can be mediated by cytokine-induced activation of the PI-3K and protein kinase-Cζ (PKCζ) -axis (43). This leads to a model were the function of FcαRI is actively suppressed by phosphorylation by GSK-3 in unactivated cells. Cytokine-induced activation of PI-3K followed by activation of PKCζ leads to phosphorylation and inactivation of GSK-3. This in turn leads to dephosphorylation and activation of FcαRI. It is still unclear at which level the control of dephosphorylating of the receptor is achieved. It might be that a constitutive active phosphatase dephosphorylates the receptor or that such enzyme is actively controlled by inside out signals such as found for FcγRI (39). Unfortunately, a similar concept has not been developed in any detail for the inside-out control of FcγRII.

### Mechanism of Inside-Out Control of FcγRI (CD64) Functionality: The Tail and Importance of Phosphatase Activity

The situation is different with FcγRI as this is a high affinity receptor able to bind to monomeric IgG. The general idea is that this receptor is always occupied by ligand under conditions such as found in the plasma. Nonetheless, several indications in studies by the group of Leusen et al. (55) provided evidence that this receptor irrespective of bound monomeric IgG can still bind to immune complexes. Only this latter binding is sensitive for inside-out control. The receptor ectopically expressed in hematopoietic cell lines is sensitive for inside-out signaling. The concept arising from this study is that the phosphatase PP2A is the driving enzyme involved in dephosphorylating the receptor and thereby activating its functionality (39). Here again the phosphorylated receptor has a low functionality and dephosphorylation leads to activation. The underlying mechanisms are not yet completely understood, but recently Brandsma et al. have described that inside-out control of FcγRI is at least in part mediated by lateral movement of the receptor in the membrane (54). It is tempting to speculate that this modulation of movement will be important for the control of valency of this receptor.

### The Intracellular Tail and the Inside-Out Control of FcγRII (CD32)

The importance of the intracellular tail of FcγRII comes from experiments in cell lines showing that ectopically expressing tail-less version of FcγRIIA/B is accompanied by a blunted signaling response (17). To test the hypothesis that the tail of FcγRIIA is also important in phagocytes Clark et al. (56) transduced neutrophils with a cell permeant peptide encompassing the intracellular tail of FcγRIIA. They could show that this peptide decreased Ca^2+^ signaling as well as formation of phagolysosomes in human neutrophils.

It is clear that the intracellular tails of FcγRIIA/B, FcγRIIIA, and FcαRI are important for signaling. However, tail-less mutants co-expressed with other receptors such as integrins are still able to transmit signals indicating the intimate cross-talk between these receptors and alternative signaling chains (57).

## Inside-Out Control and Receptor Interactions

The view that only valency and affinity are important for the inside-out function of Fc-receptors is too simple. The complexity of the ligands (uni/multi valent, fixed complement etc.), immune complexes and opsonized microbes, is very relevant. Here additional proteins and other ligands are present/expressed that can bind to a multitude of additional receptors on innate effector cells e.g., integrins, Toll-like receptors, glucan receptors, complement receptors etc. It is to be expected that differential inside-control mechanisms will control some if not all of these receptors. It will be clear that the net result of all these interactions will lead to a very complex situation that is difficult to understand from the view of individual receptor function.

### FcγR/FcγR Cross-Talk

Most innate effector cells express multiple FcRs and most multiple FcγR's. Monocytes and macrophages express FcγRI, FcγRII, and FcγRIII, neutrophils FcγRIIA and FcγRIIIB. Eosinophils only express FcγRIIA and maybe FcγRIIB/C. Cross-talk between FcγRII and FcγRIIIA/B has been suggested by various experiments. Co-crosslinking of FcγRIIA and FcγRIIIB leads to a clear activation of neutrophils characterized by changes in [Ca^2+^]_I_ and downstream functions (29). On NK-cells FcγRIIA and FcγRIIIA cross-modulate their functions (58). It is tempting to speculate that subtle changes in inside-out control of these individual receptors will influence the end result of co-activation.

### FcR-Integrin Cross-Talk

Several studies have shown that the interaction of primary cells expressing both integrin receptors and FcRs with opsonized targets is characterized by a clear cross-talk (30, 59–61). Again this is best shown in cells that express relatively little different FcRs to exclude interference of above mentioned FcγR/FcγR interactions. Again eosinophils are an interesting cell model as they only express FcγRIIA as activating FcγR. It has clearly been shown that a synergism is present when a surface is expressing integrin ligands such C3bi (ligand of Mac-1/CR3) together with Ig's. Van der Bruggen et al. have shown that yeast opsonized with both ligands is superior when compared with yeast only coated with Ig's or complement (60). However, a trivial explanation might be that the affinity/avidity of the opsonin receptors might be higher when both ligands are present.

Ortiz-Stern et al. have described the importance of cross-linking of FcγRIIIB on neutrophils in modulation of β1-integrins whereas cross-linking of FcγRIIA and FcγRIIIb both lead to activation of β2-integrins (62). More of this type of cross-talk between FcRs and integrins has been reviewed by this group (59). Relevant for this concept is the finding that a genetic polymorphism in the FcγRIIIb gene affects the interaction of this receptor with FcγRIIA and Mac-1/CR3 (CD11b/CD18) (63).

### FcR-TLR Cross-Talk

Apart from functional interactions between opsonin and integrin receptors, the function of FcRs is also modulated by multiple other receptors. An important class are the pattern recognition receptors such as toll-like receptors. These receptors can engage with FcR signaling by physical interaction as well as through signaling after ligand binding (64). Indeed, co-immunoprecipitation studies in murine neutrophils have shown that TLR4 (LPS-receptor) physically interacts with FcγRIII upon binding to its ligand LPS (64). It is good to emphasize that there are marked differences in FcRs and Toll-like receptors between mouse and man (4, 65).

For cross-talk between FcR and TLR both receptors do not need to physically interact as the main signaling pathways induced by TLR activation, NFκb, MAP-kinases and PI-3 kinase, are important in inside-out control of FcγRIIA and FcαRI. More of these interactions between FcR and TLR have been excellently reviewed recently (66).

### Inside Control of FcRs by Other Receptors or Signaling Molecules

Apart from TLR's there is a whole range of cytokine/chemokine receptors and glucan receptors that all have in common that they engage in signaling pathways important for inside-out control of FcRs. It will be clear that these signals control the interaction between innate effector cells and their targets. This mechanism has been known for a long time and was generally referred to as priming: a process that does not induce a certain cell function by itself but greatly enhances this response to a (heterologous) agonist (67). Particularly, cytotoxic responses are sensitive for such priming responses that act as “safety locks” to prevent aspecific activation of inflammatory cells. Part of such a priming response is mediated by the interaction of FcRs with function modulating membrane proteins.

Not many membrane receptors/chains other than FcR-γchain, additional FcRs or integrins have been described to be involved in the functionality of FcRs. The correct expression of FcRs is dependent on the presence of β-2 (CD18) integrins. Kindzelskii et al. have described the aberrant capping responses of membrane proteins including FcγRIII and the urokinase receptor in patients with leukocyte adhesion deficiency I (LADI) (68). The data imply that a physical cross talk between integrins and FcRs is part of the correct functioning of FcRs (57). The reverse has not been published.

Next to these aforementioned binding partners periplakin has been implicated in the regulation of function of FcγRI (69). The authors described that periplakin was important in receptor recycling as well as ligand affinity. Periplakin has also been implicated in the control of G-protein coupled receptors, which might important for the signaling of FcR in trans (70) (see below).

## Inside-Out Control and Glycosylation of Fc-Receptors and Immunoglobulins

In recent years another concept of inside-out control has emerged. It turned out that differences in glycosylation of Fc-receptors has a major impact on their functionality as has recently been reviewed by Hayes et al. (71). This mode of control is nicely illustrated by Patel et al. (72) showing that the function of FcγRIIIA on NK-cells is dependent on its glycan composition. This implies that post-translational processing of FcR is of importance for their functionality on the cell membrane (73). It is not only the glycosylation of FcRs that is important, but also the glycosylation of the different Ig's as large differences are found between the functionality of certain Ig's depending on their N-glycan content (74, 75). Interestingly, also anti-inflammatory characteristics of IgG can be attributed to differences in glycosylation (76). In conclusion, by affecting glycosylation of both FcRs as well as Ig's immune cells can steer the immune response. This has major consequences for designing therapeutical antibodies (77).

## Inside-Out Control and Signaling in *Trans*

As mentioned before FcRs can signal through their intracellular tail and/or through an accessory FcRγ chain constitutively associated with the receptor. A third mechanism is activation in trans through heterologous receptors associated with the FcRs only after (pre)activation. This concept of signaling in *trans* has been identified many years ago for signaling through G-protein coupled receptors directly activating growth factor receptors such as the EGF receptor (78). This mode of transactivation between receptors seems important for FcRs. Several interesting interactions have been published.

### FcRs and Other FcRs

Most of the data regarding transactivation of FcRs to other FcRs is indirect. Nevertheless, several lines of evidence show that co-crosslinking of different FcRs leads to differences in signaling. Vossebeld et al. showed that co-crosslinking FcγRII and FcγRIII lead to more mobilization of intracellular free Ca^2+^ (29). This study also implied a function for FcγRIII as this PI-linked protein was still able to modulate signaling through FcγRIIA. Other studies have shown that cross-linked FcRs lead to differences in the activation of the MAPkinase signaling pathways (20, 79). Interestingly, co-crosslinking of FcRs leads to differential of adhesive phenotypes dependent on the type of FcR and their polymorphisms (80). This mechanism might be important in the fine tuning of responses of leukocytes with different immune complexes. A next level of complexity comes from studies showing functional antagonistic behavior of FcγRIIA and FcγRIIIB (81). These authors provided evidence that immune complexes that are endocytosed by FcγRIIIB are cleared that is considered as anti-inflammatory whilst this process mediated by FcγRIIA leads to Netosis that is considered to be pro-inflammatory. These studies imply that subtle changes brought about by inside out signaling determines the type of the immune response.

### FcRs and Integrins

Most data on FcR signaling in *trans* is through integrins. Many studies imply that FcRs pair with different integrins upon activation with immune complexes or by crosslinking of the receptors by anti-receptor antibodies. However, these experiments in primary cells that cannot be genetically manipulated are difficult to interpret in terms of receptor specific signaling as there will be interplay between these receptors, and other modulating membrane receptors where it is basically impossible to determine which signal originates from which signaling chain. To circumvent these “chicken and the egg” issues experiments have been performed in cell lines ectopically expressing FcRs and integrins. Poo et al. have described the physical interaction between FcγRIII and Mac-1 (CD11b) in fibroblasts (82). A similar finding described the interaction between FcγRII and Mac-1 (57). This latter interaction is important for FcγRII mediated phagocytosis. Indirect experiments show that these interactions are also important in the response of neutrophils with opsonized particles (83). The concept that Mac-1 can transduce signals for other Mac-1 binding partners has been described before (84).

### The Interaction Between FcRs and G-Protein Coupled Receptors (GPCR)

The interaction between FcRs and G-protein coupled receptors (GPCR) can cross regulate their functions. It has been established that the function of FcγRII on eosinophils is upregulated by priming evoked by agonists of GPCR (67). However, it is uncertain whether a physical interaction between FcγRII and GPCR is necessary or that the activated GPCR activates the receptor by cytosolic signaling. Relevant is, however, that periplakin that regulates the functionality of FcγRI (CD64) can also bind GPCR's (70) supporting a potential bridging role of periplakin between FcRs and GPCR's. Such functions have been amply described in the control of integrins, which has been recently reviewed by Ye et al. (38).

### FcRs With Other Proteins

FcRs with other proteins have been described but one should be aware of the fact that the intimate interaction between integrins and FcRs might preclude the identification of other binding partners: in multimolecular complexes these proteins such as integrin associated protein (85) or thrombospondin (86) might bind to integrins rather than the associated FcR.

## Inside-Out Control, Membrane Domains, and Lateral Movement

Up to now the functionality of FcRs has been described as if the receptors are free flowing in the plane of the plasma membrane. This is, however, a too simple view as the membrane is organized in domains with different fluidities. Best studied are the micro domains rich in cholesterol also referred to as lipid rafts (87). But other specialized domains such as found in the lamellipodium (88) and uropods (89) are also characterized as being enriched in important receptors and signaling molecules. Receptors can therefore be localized at different membrane compartments that are relatively slowly interacting. Not much is known regarding the distribution of FcRs in these different domains, but recent studies support the importance of lateral mobility of FcRs in the plain of the membrane and the importance of co-localization in these domains (54). In addition, Ten Broeke et al. provided evidence that dephosphorylation of FcαRI and functional activation of the receptor is associated with enhanced lateral movement of the receptor and possibly an increase in valency of the receptor (43). Moreover, data of Yang et al. implied that cross-linking of FcγRIIIb (CD16b) leads to lipid raft mediated activation of SHP2 (51).

## Inside-Out Control and the High Affinity Receptor for IgE, FcεRI

The main emphasis in this review was inside-out control of IgG and IgA receptors as this process was best described in this context. However, several studies clearly indicate that also the function of FcεRI is controlled by inside-out signals. This control has been excellently reviewed by Kraft and Kinet (90). Important for this review is the requirement of expression of the tetraspanin CD63 for optimal functionality of FcεRI on mast cells (91). As CD63 is expressed in granules this finding links degranulation with an optimal function of FcεRI. Several other processes are involved in the control of FcεRI by either activating (92) or inhibiting the receptor (93). These processes are now seen as therapeutic targets in allergic diseases (6).

## The Implications of Inside-Out Control in Clinical Applications of Humanized Antibodies

The implications of FcR inside-out control for the treatment of patients with clinical humanized antibodies are just emerging. The approach will obviously depend on the requirement of effector cells in such therapy and the FcR that they express. Treatment with blocking antibodies directed against single molecules (such as cytokines, complement fragments, and chemokines) might not be directly affected by inside-out control of FcRs as these receptors do not have an obvious role here. However, FcRs play a role in clearance of these target-antibody complexes as the majority is cleared by endocytosis and will subsequently be degraded in the lysosomal compartment (53). This may indicate that therapeutic antibodies might be more rapidly cleared in patients with inflammatory diseases that are characterized by the presence of priming mediators in the peripheral blood or tissue (94). Under these conditions inhibition of inside-out control might be a therapeutic target as it might preserve therapeutic doses of these antibodies allowing lower dosing of the antibodies.

The situation with several antibodies might be more complex. Particularly, those antibodies blocking the function of cellular receptors are of interest. On the one hand, one might want to inhibit inside-out control for preservation of sufficient therapeutical concentrations (see above) on the other inside-out activation might be beneficial for the clinical effect. The idea behind this conception is the following. Anti-receptor antibodies or antibodies directed against cell bound cytokines not only block these molecules, but they might also enable the cell expressing these proteins to be killed (95). This is mediated by antibody or complement dependent cytotoxicity: ADCC or CDC, respectively. Binding of antibodies and/or complement to cells leads to opsonization. Phagocytic receptors are particularly directed against multivalent ligands such as a surface covered with antibodies or complement. The phagocytes will then activate the same armamentarium normally employed for the killing of micro-organisms. The result is a cytotoxic response toward the opsonized cell instead of micro-organism. As both complement receptors such as complement receptor 3 (CR3/Mac-1/CD11b) and FcRs such as FcγRIIA (CD32) and FcαRI (CD89) are very sensitive for inside-out activation it will be clear that this activation is very important for the clinical action (53, 84). Not much is known regarding these issues in humans *in vivo* some studies now imply that ADCC is often important for the clinical effect of therapeutic antibodies (96, 97). A clear example is the anti-IL5R antibody, Benralizumab, which functions through ADCC (95) of IL5Rα+ cells [eosinophils, basophils and possibly ILC2 (98)]. The concept of inside-out activation of the ADCC under these conditions has not been applied to these clinical studies.

The overall conclusion whether or not inside-out control should be considered in augmenting the therapeutic is likely to be dependent on the mode(s) of action of the therapeutic antibodies. It is, however, clear that this complexity should be considered gaining optimal therapeutic effectiveness of current and new antibodies.

## Conclusion

Inside-out control of FcRs as well as integrins functions as a safety lock preventing collateral damage evoked by innate immune effector cells. Here a clear cross-talk is present between the adaptive immune response producing priming mediators and the innate immune system that adapt to these signals. Part of the priming mediators liberated during inflammation leads to inside-out control of FcRs potentiating these receptors. This very complex mechanism is based on modulation of valency of the receptors, their affinity, their interaction with other signaling chains and receptors and their localization in specialized membrane areas such as lipid rafts. The many levels of control will make it possible to fine tune the inside out control with therapeutic molecules only affecting part of this process. This will allow stratified therapy such that the therapeutic effect is maximal while the normal function of phagocytes is preserved.

## Author Contributions

The author confirms being the sole contributor of this work and has approved it for publication.

### Conflict of Interest Statement

The author declares that the research was conducted in the absence of any commercial or financial relationships that could be construed as a potential conflict of interest.
