# Corrigendum: Inside-Out Control of Fc-Receptors

**DOI:** 10.3389/fimmu.2019.00971

**Published:** 2019-05-07

**Authors:** Leo Koenderman

**Affiliations:** Department of Respiratory Medicine and Laboratory of Translational Immunology, University Medical Center Utrecht, Utrecht, Netherlands

**Keywords:** inside-out control, immunoglobulins, priming, activation, phagocytes, Fc-receptors

In the original article, there was an error. Tyrosine-phosphatases were incorrectly referred to as tyrosine-kinases.

A correction has been made to the following:

Section: Signal transduction, page 2

Subsection: Direct signaling, paragraph 1

Corrected paragraph:

Direct signaling by CD32 is mediated by immunoreceptor tyrosine-based activation motif (ITAM/CD32A and CD32C) (17) and by immunoreceptor tyrosine-based inhibitory motif (ITIM/CD32B) (18). These motifs determine whether the receptors are activating or inhibitory. It is important to emphasize that signaling starts by cross-linking of the receptor leading to activation of phosphatases such as SHP and SHIP, and members of the src-family of tyrosine kinases (19–21). This leads to phosphorylation of the important tyrosine residues in the ITAM/ITIM motifs from where various signaling cascades are initiated. Phosphorylation of ITAMs lead to activation of the cells (22), whereas phosphorylation of ITIMs lead to cell inhibition (23). The mechanisms involved in the control of CD32B have been excellently reviewed by Getahun and Cambier (24).

The author apologizes for this error and states that this does not change the scientific conclusions of the article in any way. The original article has been updated.

